# Recent advances in CRISPR-based genome editing technology and its applications in cardiovascular research

**DOI:** 10.1186/s40779-023-00447-x

**Published:** 2023-03-10

**Authors:** Zhen-Hua Li, Jun Wang, Jing-Ping Xu, Jian Wang, Xiao Yang

**Affiliations:** 1 State Key Laboratory of Proteomics, Beijing Proteome Research Center, National Center for Protein Sciences, Beijing Institute of Lifeomics, Beijing, 100071 China; 2 Yaneng BIOScience (Shenzhen) Co., Ltd., Shenzhen, 518102 Guangdong China

**Keywords:** Genome editing, CRISPR-Cas system, Base editing, Prime editing, Transposon-associated genome editing, Cardiovascular disease, Heart, Blood vessel, Gene therapy

## Abstract

The rapid development of genome editing technology has brought major breakthroughs in the fields of life science and medicine. In recent years, the clustered regularly interspaced short palindromic repeats (CRISPR)-based genome editing toolbox has been greatly expanded, not only with emerging CRISPR-associated protein (Cas) nucleases, but also novel applications through combination with diverse effectors. Recently, transposon-associated programmable RNA-guided genome editing systems have been uncovered, adding myriads of potential new tools to the genome editing toolbox. CRISPR-based genome editing technology has also revolutionized cardiovascular research. Here we first summarize the advances involving newly identified Cas orthologs, engineered variants and novel genome editing systems, and then discuss the applications of the CRISPR-Cas systems in precise genome editing, such as base editing and prime editing. We also highlight recent progress in cardiovascular research using CRISPR-based genome editing technologies, including the generation of genetically modified in vitro and animal models of cardiovascular diseases (CVD) as well as the applications in treating different types of CVD. Finally, the current limitations and future prospects of genome editing technologies are discussed.

## Background

Genome editing technology refers to a series of technologies capable of manipulating cellular DNA sequences at desired genomic sites by generating altered DNA sequences through nuclease-mediated site-specific DNA breaks that are resolved through DNA repair pathways [1–3]. Among genome editing-associated nucleases, clustered regularly interspaced short palindromic repeats (CRISPR)/CRISPR-associated protein (Cas) nucleases are convenient, efficient, and precise, and are currently the most widely used [4–8]. After the CRISPR-Cas9 system was characterized and programmed to perform RNA-guided DNA cleavage at specific sites in prokaryotes, it was immediately proven to be an efficient tool for editing eukaryotic genomes [9]. Since then, CRISPR-based genome editing technology has drawn a worldwide attention and initiated extensive development. Emerging CRISPR-based tools with broadened targeting ranges, improved editing specificity and efficiency, and other distinct capabilities have facilitated eukaryotic genome editing by selecting optimal CRISPR-Cas tools. In addition to expanding the CRISPR-Cas nuclease arsenal, this system has also been applied to transcriptional regulation, epigenetic modification, and live-cell imaging by incorporation with other effector proteins [7, 10, 11].

The exponential development of genome editing technology has dramatically changed the landscape of biological and medical research, heralding a new era of precision medicine based on genome editing [12]. CRISPR-based nucleases are able to cut target DNA and generate double-strand breaks (DSBs), followed by the introduction of random mutations mediated by non-homologous end-joining (NHEJ), or make precise editing through homology-directed repair (HDR) [13]. The therapeutic potential of CRISPR-based tools has been investigated using the mouse models of various human diseases [7, 14]. However, precise gene correction for in vivo therapeutic utility remains challenging, which is partially due to the low efficiency of HDR-mediated DNA replacement. This strategy is usually not applicable to post-mitotic cells, as HDR occurs mainly in the S/G_2_ phase during cell division [15]. Precise genome editing tools have been developed and continuously optimized by fusing activity-impaired Cas nucleases with deaminases, called base editors, or with reverse transcriptases, called prime editors (PEs) [16–18]. Despite not achieving the goal of arbitrarily introducing any genetic substitutions at any targeted genomic site, we are now closer to this aspiration than ever.

Cardiovascular disease (CVD) is a group of disorders of the heart and blood vessels that has consistently been ranked as the leading threat to human health worldwide. Many gene mutations have been linked to CVD, and the number is still increasing [19, 20]. Loss-of-function studies in animals are required to address the causal relationship between these mutations and cardiovascular pathologies. With the help of the CRISPR-based toolbox, creating animal models of human diseases has become much easier, faster, and more flexible than ever before; these models will greatly advance our understanding of cardiovascular pathogenesis and the development of therapeutic strategies [14]. Furthermore, CRISPR-based genome editing technology holds promise for treating inherited CVD caused by rare mutations.

In this review, we highlight the recent advances in CRISPR-based genome editing technology, mainly in the past three years, and discuss the tremendous innovation this epoch-making technology has brought to the field of cardiovascular research.

## Novel Cas orthologs and engineered variants

**Fig. 1:**
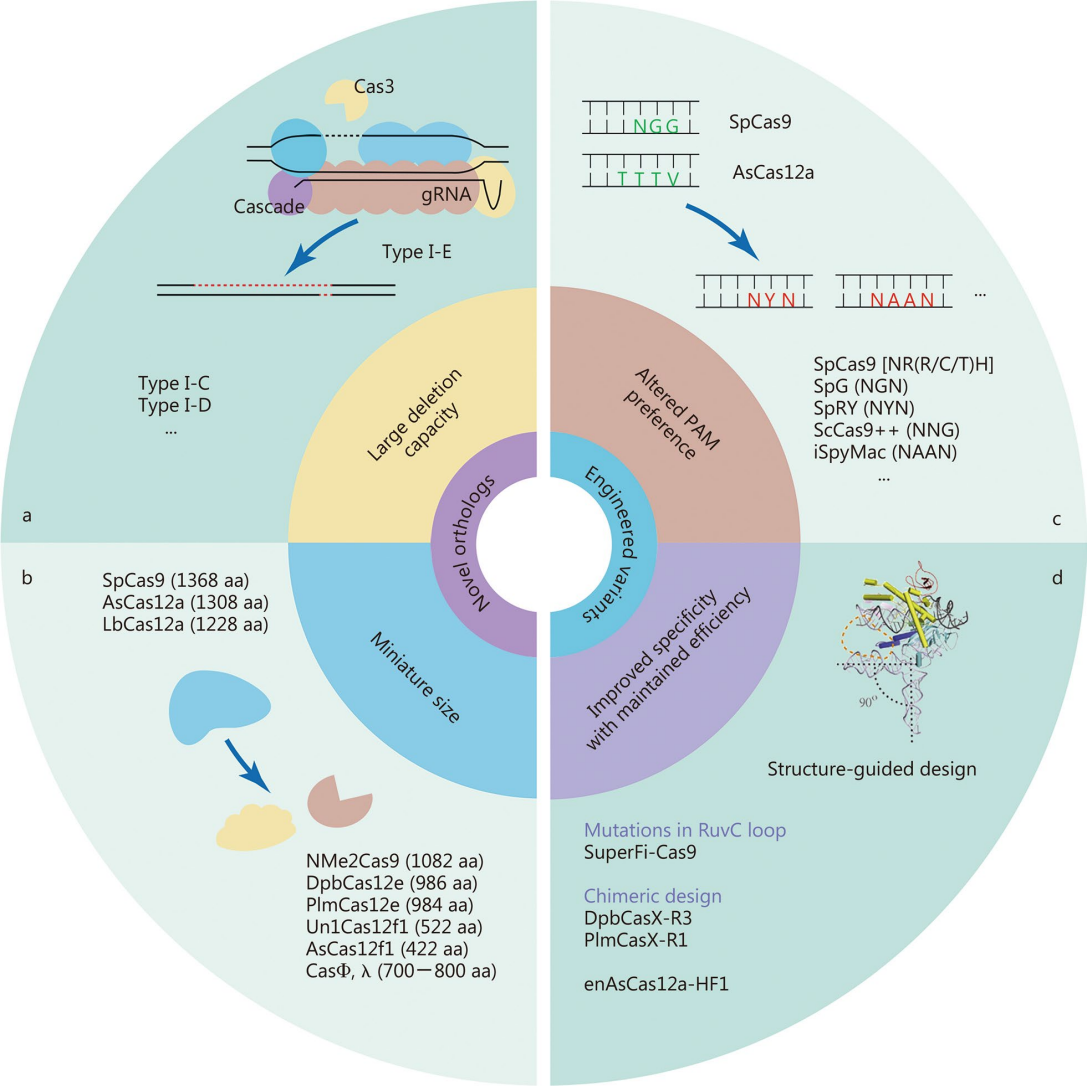
Characteristics of novel Cas orthologs and engineered variants. **a** Representative type I Cas orthologs capable of large-range deletions. **b** Representative Cas orthologs of miniature sizes. **c** Engineered Cas variants with diverse protospacer adjacent motif recognition capabilities. **d** Structure-guided strategies for improving DNA specificity without affecting the on-target cleavage efficiency

Natural CRISPR-Cas systems are originally identified as adaptive immune systems in bacteria and archaea, and can be divided into two classes based on their composition and mechanisms. These systems are further divided into six types (I–VI) and dozens of subtypes based on the characteristics and accessory genes flanking the CRISPR array [21]. The most widely used class 2 CRISPRs are characterized by their single effector proteins, including type II Cas9 and type V-A Cas12a. Although class 2 natural Cas nucleases have long been used for efficient genome editing, their applications are limited because of the requirement of specific protospacer adjacent motif (PAM) sequences, off-target DNA cleavage, and occasionally, large sizes. Class 1 CRISPR systems possess multiple effector molecules that have unique features, such as distinct PAM preferences, higher on-target specificity through longer target recognition, and production of long-range genomic deletion [22–25]. However, the requirement of multiple effectors and the relatively low editing efficiency must be improved before their widespread application. Continuous efforts have been made to characterize novel Cas orthologs and engineered Cas variants to improve genome editing efficiency and broaden compatibility (Fig. 1).

### Characterizing novel Cas orthologs with distinctive features

The class 1 type I CRISPR system is the most prevalent CRISPR system, in which the multi-subunit CRISPR-associated complex for antiviral defense (Cascade) identifies DNA targets, and the helicase-nuclease enzyme Cas3 degrades DNA [26] (Fig. 1a). Several type I CRISPR systems have been characterized and applied to mammalian genome editing. Type I-E and type I-D systems have been used to induce unidirectional and bidirectional long-range deletions in human cells [22–24]. Recently, supplying Cas11 was shown to enable divergent I-C, I-D, and I-B CRISPR-Cas3 editors for eukaryotic applications, and efficiently produced large unidirectional deletions [25]. Therefore, type I CRISPR systems can greatly expand the genome editing toolbox owing to their unique mechanisms and advantages in deleting full-length genes, gene clusters, and non-coding sequences.

Recently, the IS200/605 transposon family encoded RNA-guided nucleases have been identified as ancestors of CRISPR-Cas nucleases [27, 28]. Cas9 endonucleases could likely have evolved from ancestral IscB proteins, whereas Cas12 endonucleases descended from TnpB proteins [27]. These transposon-encoded nucleases, together with the IsrB proteins, which are shorter IscB homologs also encoded in IS200/605 superfamily transposons, are called the obligate mobile element-guided activity (OMEGA) system [27]. IscB and TnpB are guided by non-coding RNAs called ωRNAs, which are derived from the left- or right-end elements of a transposon and combine the functions of CRISPR RNA (crRNA) and trans-activating CRISPR RNA (tracrRNA) [27–29]. Being only two-fifths the size of Cas9, IscB and TnpB can mediate double-strand DNA cleavage at the target sites with a 3′ or 5′ transposon-associated motif (TAM), and both have been adopted for genome editing in human cells [27, 28].

The in vivo application of most CRISPR systems is challenging because of their large size, especially when delivered by the widely used adeno-associated virus (AAV). As a result, the exploitation of miniature Cas proteins with high efficiency is in sustained demand (Fig. 1b). For instance, SaCas9 (1053 aa), CjCas9 (984 aa), and Nme1Cas9 (1082 aa) have been validated as mammalian genomic editors [30–32]. Recently, a compact Nme2Cas9 (1082 aa) recognizing an N_4_CC PAM was described with an identical target density as SpCas9 and few off-target effects [33]. In addition to the Cas9 orthologs mentioned above, several Cas12 nucleases with smaller sizes, such as Cas12e (or CasX, 986 aa), and Cas12f (400–700 aa), have also been identified as genome editing tools in mammalian cells [34–37]. The Un1Cas12f1 (522 aa) system was optimized to enable efficient genome editing in human cells [35]. Notably, with only a size of 422 aa, AsCas12f1 is currently the smallest RNA-guided Cas nuclease, and has been shown to be an effective programmed genome editing tool in both bacterial and human cells [36]. With approximately equal to or less than half the size of the widely used SpCas9, these miniature CRISPR tools facilitate the AAV-mediated all-in-one delivery of CRISPR components or catalytically inactive Cas variants fused with other functional proteins.

Very recently, CRISPR-Cas systems were found to be widely encoded in the genomes of diverse bacteriophages, where they are involved in competition with other viruses [38, 39]. The bacteriophage-encoded Cas proteins contain all known types of CRISPR-Cas systems, but have phage-specific properties. These Cas proteins, such as CasΦ [38] and Casλ [39], tend to have remarkably small sizes due to the compact viral genome. These hypercompact systems have been shown to edit the genomes of human and plant cells, indicating that viral Cas nucleases could serve as a new source of genome editing tools.

### Expanding the range of genomic targets

Genomic targeting by Cas nucleases requires a PAM sequence near the site where the Cas nuclease binds DNA sequences complementary to the single guide RNA (sgRNA). The requirement of PAM is the gatekeeper for CRISPR-Cas mediated genome targeting, as whether a genomic sequence possesses a PAM for a certain Cas nuclease determines whether the site can be targeted and edited by CRISPR. Efforts have been made to develop Cas nucleases with broader PAM compatibility to pursue true PAM-free nucleases. However, PAM-free nucleases might have potential drawbacks, such as self-targeting of gRNA-expressing DNA constructs and reduced efficiency as more time is required for interrogating the whole genome. Therefore, it is better to develop an arsenal of divergent PAM-dependent Cas nucleases that collectively cover all genomic sequences [40] (Fig. 1c).

Using phage-assisted non-continuous and continuous evolution strategies, three new SpCas9 variants (SpCas9-NRRH, SpCas9-NRCH, and SpCas9-NRTH) were characterized to recognize most NR PAM sequences, together with SpCas9-NG (N = A/T/C/G, R = A/G, H = A/C/T) [41]. To relax the PAM preference of SpCas9, two SpCas9 variants, SpG (targeting NGN) and SpRY (targeting NYN), have been generated by structure-guided substitutions in several residues, making most of the genome targetable (Y = C/T) [42]. Structure-motivated engineering has also been used to expand targeting range of LbCas12a and AsCas12a [43, 44]. Chimeric Cas proteins created by exchanging PAM-interacting domains between naturally occurring Cas orthologs have also been applied to expand PAM recognition. Substituting the loop sequence of Cas9 from *Streptococcus anginosus*, together with the T1227K mutation, into the open reading frame (ORF) of ScCas9 generates ScCas9++ with NNG PAM compatibility [45]. A similar strategy was used to generate a variant iSpyMac by grafting the PAM-interacting domain of SmacCas9 into SpyCas9, which recognizes all adenine dinucleotide PAM (NAAN PAM) sequences [46]. The chimera generation approach has also been applied to replace PAM-interacting domains in SaCas9 [47] and Cas12a [48].

### Improving the DNA specificity without affecting on-target cleavage efficiency

Off-target activity is a major challenge when using CRISPR tools for disease-related gene therapy. Over the years, continuous efforts have been made to construct high-fidelity Cas9 variants including eSpCas9(1.1) [49], SpCas9-HF1 [50], HypaCas9 [51], evoCas9 [52], HiFi Cas9 [53] and Sniper-Cas9 [54]. In addition, high-fidelity SaCas9s have been identified either by rational engineering [55] or directional screening [56]. The achievement of enhanced discrimination between on-target and off-target binding of these variants relies mainly on the energetic destabilization of the Cas9:sgRNA:DNA complex at off-target sites [57]. However, the improvement of these high-fidelity Cas9 variants seems to occur at the cost of decreased on-target efficiency [58, 59]. Recently, kinetics-guided cryo-electron microscopy was used to show that mismatches distal to PAM can be stabilized by a loop in the RuvC domain, allowing Cas9 activation [60]. Based on this observation, they designed a high-fidelity variant with mutations in the RuvC domain, named SuperFi-Cas9, which displayed significantly improved mismatch discrimination without compromising on-target DNA cleavage efficiency [60].

DpbCas12e has been validated as a naturally occurring high-fidelity Cas nuclease with striking avoidance of off-target activity [61]. In a recent cryo-EM-based structural engineering study, unique nucleotide-binding loops within Cas12e were found to be important for DNA cleavage efficacy. Based on this finding, newly designed chimeric Cas12e proteins (DpbCasX-R3 and PlmCasX-R1) and sgRNA (sgRNAv2) exhibited substantially improved DNA editing efficiency in mammalian cells [62]. Structure-guided protein engineering has also been used to improve the performance of AsCas12a [43]. E174R/S542R/K548R substitutions were introduced into AsCas12a to construct a variant called enAsCas12a that possesses an expanded targeting range and increased cleavage activity. A high-fidelity version of enAsCas12a (enAsCas12a-HF1) with an additional N282A substitution was also engineered to reduce off-target effects [43] (Fig. 1d).

## Advances in precise genome editing

**Fig. 2:**
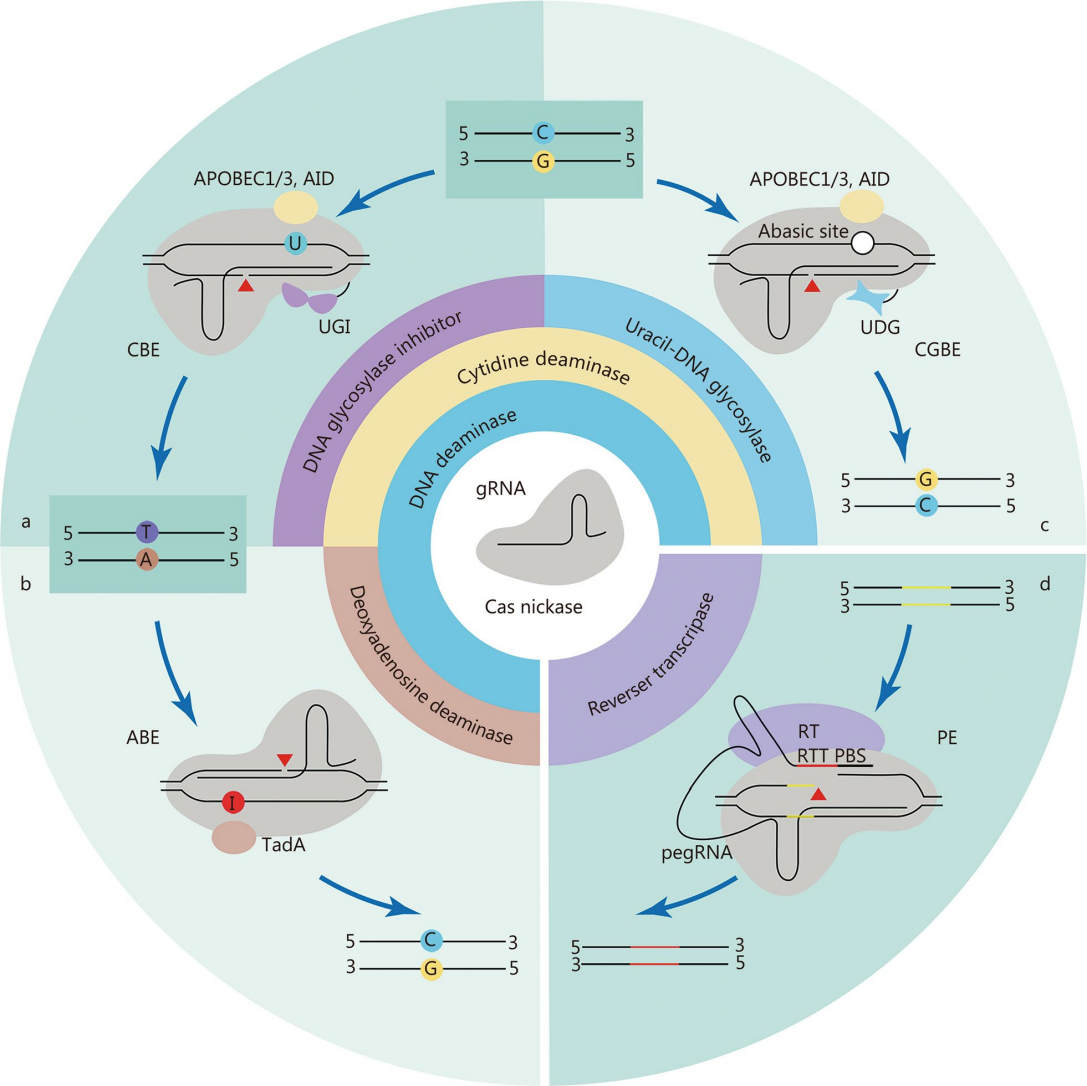
CRISPR-Cas-based DNA base editing tools. **a-c** Schematic diagrams of CBE (**a**), ABE (**b**), and CGBE (**c**). **d** Schematic of PE. UGI uracil-DNA glycosylase inhibitor, AID activation-induced cytidine deaminase, UNG uracil-DNA glycosylase, TadA deoxyadenosine deaminases, RT reverse transcriptase, PBS primer binding site, RTT RT template, CBE cytosine base editor, ABE adenine base editor, CGBE C-to-G base editor, PE Prime editor

Precise genome editing is essential for preclinical research and clinical gene therapy, and HDR-mediated gene editing has long been the only option. Efforts have been made to improve HDR efficiency, such as the use of rationally designed single-stranded oligodeoxynucleotide (ssODN) templates instead of double-stranded DNA [63] or the addition of NHEJ chemical inhibitors [64]. The delivery of Cas9 and HDR templates by AAVs has accomplished precise genome editing in post-mitotic hippocampal neurons and cardiomyocytes in mice [65–67]. However, the efficiency of HDR-mediated editing is still relatively low compared to that of the predominant NHEJ repair pathway, and DSBs made by this conventional method may introduce undesired damage to the genome [68–70]. Its clinical application is hampered by the need for additional DNA templates and HDR-promoting chemical agents with potential cytotoxicity, such as SCR7 [64], azidothymidine, trifluridine [71], NU7026, and NU7441 [72]. Motivated by these problems, novel precise genome editing tools that do not require DSBs or exogenous DNA templates have been developed (Fig. 2).

### Base editors

Base editors can make precise base substitutions without requiring DSBs or donor DNA templates, and are independent of HDR, providing a promising therapeutic tool for human genetic diseases in which the most relevant variants are single nucleotide mutations [73, 74]. Current base editors are constructed by fusing DNA deaminase enzymes to catalytically impaired Cas nucleases, which can precisely change a single base in a targeted sequence [75]. Base editing jointly harnesses the genome-targeting function of a Cas protein and the DNA base modification role of a deaminase, and sometimes additional regulatory elements are also required to achieve the desired performance. To date, base editors can be used not only for precise genome editing at specific single loci, but also for large-scale functional screening of genetic variants or key amino acid residues [76–78].

The cytosine base editor (CBE) and adenine base editor (ABE) are widely used base editors that enable the editing of all four types of base transitions (C-to-T, A-to-G, T-to-C, and G-to-A) [16, 17] (Fig. 2a, b). Since these two editors were developed in 2016 and 2017, subsequent efforts have significantly expanded their genome-targeting range and improved their efficiency and product purity. The use of Cas nickase, fusion of a second uracil-DNA glycosylase inhibitor (UGI) domain, the addition of a nuclear localization sequence, and linker and codon optimization have greatly increased the editing efficiencies of base editors [79–81]. To maximize the editing scope of base editors, diverse base editors with natural, engineered, or evolved Cas variants that recognize alternative PAMs and various deaminases have been created [82–88].

Programmable C-to-G base editors (CGBEs) that can achieve targeted C-to-G and G-to-C base transversions have recently been developed [89, 90]. CGBEs originate from CBE by replacing the UGI with a uracil-DNA glycosylase (UNG), which excises the U base generated by cytosine deaminase, resulting in an abasic site followed by the preferential installation of a G base through the DNA repair mechanism (Fig. 2c). Although CGBEs can provide efficient C-to-G and G-to-C editing, very few sites are suitable for CGBE editing. Several studies have used machine learning to optimize CGBEs to improve editing efficiency and product purity by changing the species origin, modifying the relative positions of UNG and deaminase, and optimizing codons [91, 92].

The therapeutic applications of base editors have been hampered by their genome-wide off-target effects. Recent studies have shown that cytidine deaminases used in CBE induce genome-wide off-target editing independently of sgRNA or Cas9 [93, 94]. In addition, both ABE and CBE can cause transcriptome-wide mutations [95, 96]. Continuous efforts have been made to reduce off-target effects by engineering the DNA- [97, 98] or RNA-binding domain [95, 96, 99], thereby bringing base editors closer to clinical applications.

### PEs

Prime editing is a newly developed precise genome editing technology that enables all types of base conversion, small deletions, and insertions, as desired. PEs consist of a prime editor protein and prime editing gRNA (pegRNA). The PE protein is constructed by fusing an engineered Cas9 nickase (H840A) with reverse transcriptase, which can be targeted to the genomic locus by pegRNA [18]. The pegRNA combines a gRNA recognizing the target genomic sequence, a reverse transcriptase template encoding the desired edits, and a primer binding site to initiate reverse transcription [18] (Fig. 2d). The newly synthesized edited DNA strand is incorporated into the target locus to generate heteroduplex DNA, in which the non-edited strand is eventually replaced by an edited strand through DNA repair. Compared to base editing, which often introduces bystander editing of extra bases in an activity window, prime editing is more versatile and precise.

A series of PE systems, namely PE2, PE3b, PE4, and PE5b, have been developed and are most widely used. All these systems share a common PE2 protein with an engineered Moloney murine leukemia virus (M-MLV) reverse transcriptase instead of the wild-type M-MLV reverse transcriptase in PE1 to increase editing efficiency [18, 100]. The PE3 system contains an additional sgRNA that targets the non-edited strand to increase the editing efficiency [18]. The PE2 and PE3 systems were further optimized by introducing a DNA mismatch repair-inhibiting domain MLH1dn to generate PE4 and PE5 systems, respectively [18, 100]. Systems ending in “b”, namely PE3b and PE5b, use an edit-specific nicking sgRNA to reduce indel levels [18, 100]. Constant efforts are being devoted to optimizing PEs, with a primary focus on improving their editing efficiency. Optimization of PE2 protein architecture by codon optimization, SpCas9 mutation, and alterations of the nuclear localization signal and peptide linker sequence results in PEmax protein architecture, which greatly enhances editing efficiency [100]. They also constructed two types of engineered pegRNAs (epegRNAs) by incorporating 3′ structural motifs, which stabilize pegRNA and increase prime editing efficiency [101]. Many other groups have adopted similar strategies by optimizing either PE proteins [102–104] or pegRNAs [105].

In addition, a dual pegRNA strategy has been used to improve editing efficiency, which can also achieve programmable insertion, deletion, and replacement of large genomic sequences at specific genomic sites [106–110]. Using a pair of pegRNAs, each of which targets a different DNA strand and template the synthesis of complementary DNA flaps, endogenous targeted DNA sequence between the PE-induced nick sites is successfully replaced. This strategy also achieves targeted insertion of gene-sized DNA plasmids (> 5 kb) and targeted inversions of 40 kb in human cells when co-expressing a site-specific serine recombinase, Bxb1 integrase [108]. The dual pegRNA strategy with expanded capabilities of precision genome editing provides new possibilities for treating genetic disorders caused by large DNA deletions or complex structural mutations.

## CRISPR-associated transposon (CAST) systems for large DNA insertion

CAST systems, consisting of transposase subunits and CRISPR effectors, facilitate the RNA-guided transposition of mobile genetic elements, making it a promising system for targeted, precise, and efficient insertion of large DNA segments. Most identified CASTs are derived from Tn7-like transposons that retain the core genes of the transposition machinery, but have no genes for target selection [111, 112]. Instead, CASTs co-opt nuclease-deficient CRISPR-Cas proteins to induce RNA-guided transposition [111, 112]. Several CAST systems have been experimentally or bioinformatically characterized, including type I-B, type I-C, type I-F, type IV, and type V-K CAST systems [111, 113–115]. Bioinformatic analysis of the metagenomic database also revealed a non-Tn7 CAST system that co-opts a nuclease-inactive Cas12 and type I-E cascade [111].

**Fig. 3:**
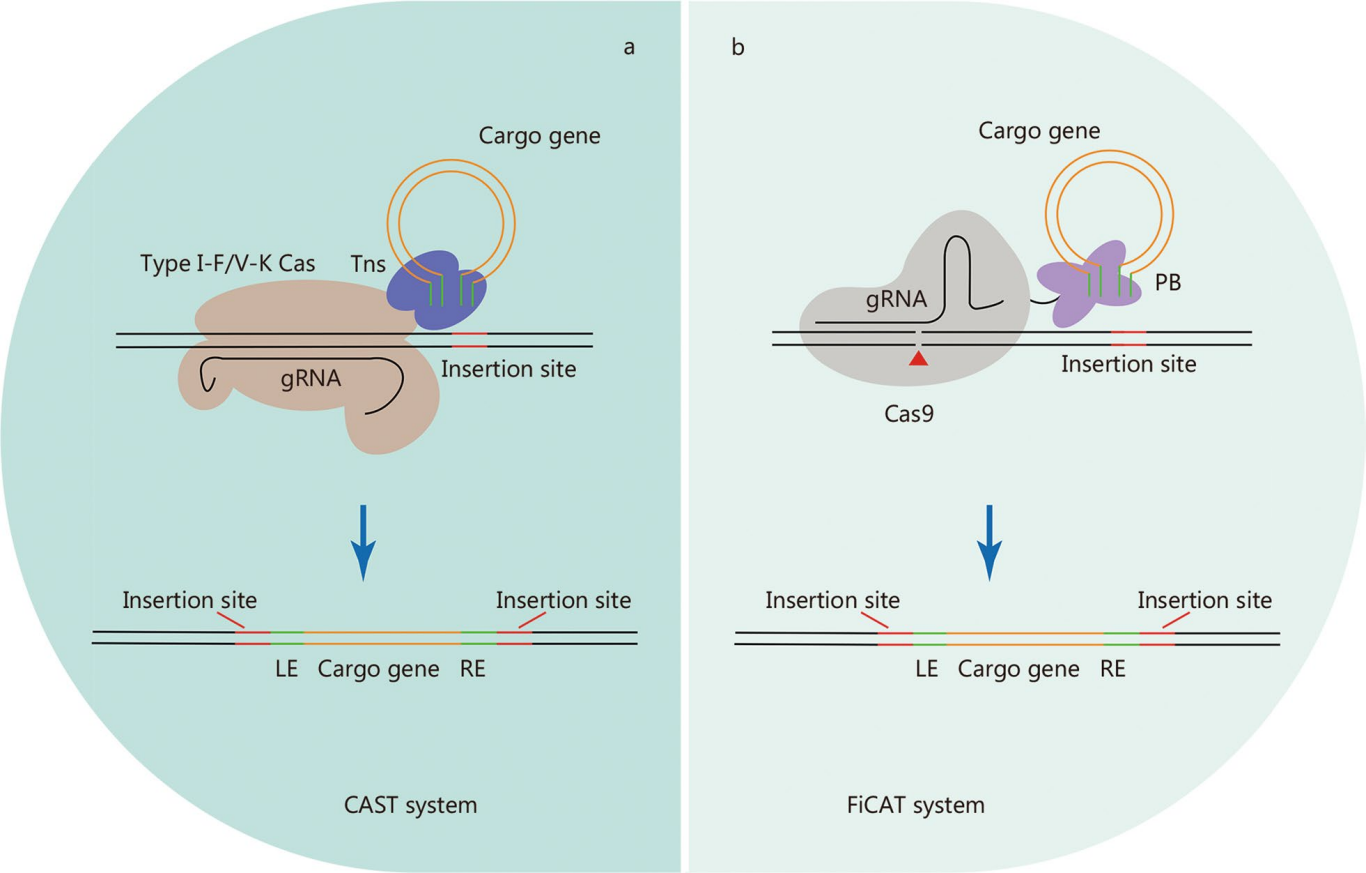
CRISPR-Cas-based transposon systems. Schematic of CRISPR-based transposon systems, CAST system (**a**) and FiCAT system (**b**), which mediate site-specific DNA integration. Tns Tn7-like transposases, PB piggyBac transposase, LE transposon left end sequences, RE transposon right end sequences, CAST CRISPR-associated transposon, FiCAT find and cut-and-transfer

Type I-F and type V-K CAST systems have been successfully reconstituted to achieve the integration of donor DNA into specific bacterial genome sites [113, 114] (Fig. 3a). An improved version of the type I-F CAST system enables highly specific and effective integration of up to 10 kb DNA fragments in the bacterial genome [116]. However, the application of these two systems in mammalian cells has not been reported. A very recent study developed an artificial transposon-associated CRISPR-Cas system named find and cut-and-transfer (FiCAT) system by coupling a SpCas9 protein with an engineered piggyBac (PB) transposase (Fig. 3b), which achieved the targeted integration of multi-kilobase DNA fragments into the genomes of mammalian cell lines and mouse liver [117]. The discovery of CAST systems has expanded the genome editing toolkit, although CAST systems still require extensive modification and optimization until they can be conveniently and effectively applied to biomedical research.

## Delivery systems for CRISPRs

The safe, effective, and tissue-specific delivery of CRISPR-Cas tools in vivo determines whether CRISPR-based gene therapy can be used for this tissue. Therapeutic in vivo delivery systems for CRISPR-Cas have recently been discussed [118, 119]. CRISPR-Cas tools can be delivered in the form of DNA, mRNA, or ribonucleoprotein complexes (RNP) through ex vivo or in vivo approaches. Various robust methods have been established to deliver genome editing reagents ex vivo, some of which have been used in multiple clinical trials involving different types of diseases [120–122]. The most efficient method of in vivo delivery of editors reported so far is the use of AAV, which can deliver editor-encoding DNA to target tissues and has been applied in clinical trials [118, 123, 124]. However, AAV-based delivery of DNA-encoding editing agents has a number of disadvantages, such as the possibility of viral vector integration into the transduced cell genome and increased frequency of off-target editing due to prolonged expression [75, 119, 125, 126], which limits its clinical application. Therefore, safer alternative strategies for in vivo delivery of genome editors must be developed.

As a gene therapy delivery system approved by the Food and Drug Administration (FDA), lipid nanoparticles (LNP) have been demonstrated to safely deliver therapeutic small molecules and nucleic acid drugs to hepatocytes and antigen-presenting cells via systemic administration or intramuscular injection. The LNP system was used to deliver gene editing tools in the first clinical trial involving human gene editing in vivo [123]. However, because intravenously delivered LNP showed liver tropism, delivering editors to non-hepatocytes has been a huge challenge. Recent studies have shown that high-throughput screening identifies nanoparticles targeting non-hepatocytes, including endothelial cells (ECs) and spleen immune cells [127, 128]. In addition, cell-type specificity of LNP-mediated Cas9 therapies could be modified by reducing Cas9-mediated insertions and deletions in hepatocytes using inhibitory oligonucleotides and siRNAs [129].

Virus-like particle (VLP) systems, which combine the advantages of viral and non-viral delivery systems, are another promising in vivo gene editing delivery vehicle [126]. VLPs can package genome editing agents in the forms of mRNA or RNP. The short cellular lifespan of RNPs effectively restricts off-target editing. Almost all current VLPs are derived from retroviruses and contain most viral components but no viral genome [118, 130–133]. Very recently, fourth-generation engineered VLPs (eVLPs) based on M-MLV have been developed to deliver Cas9 or base editor RNPs both in vitro and in vivo [126]. A single intravenous injection of eVLPs carrying a base editor targeting proprotein convertase subtilisin/kexin type 9 (*Pcsk9*) can achieve base editing in multiple tissues, reduce serum PCSK9 levels by 78%, and partially restore visual function when designed for retinal editing in a mouse model of blindness [126]. The mammalian endogenous retrovirus-like protein PEG10 has also been programmed as a VLP system called selective endogenous encapsidation for cellular delivery (SEND) platform, which can package and deliver mRNA encoding Cas9 in vivo [134]. Based on endogenous mammalian proteins, the SEND system may be less immunogenic than bona fide retrovirus-based VLP systems.

## Applications of genome editing in modeling and treating CVD

CVD, including heart and vascular diseases, are leading causes of morbidity and mortality at different ages [135, 136]. In recent years, a tremendous amount of new genetic information related to CVD has been identified using next-generation sequencing technologies [19, 20]. Owing to the advent and development of the CRISPR-Cas system, we can now handle this information and determine CVD-related functions much more easily than ever. The CRISPR-Cas system also provides more possibilities for treating inherited CVD by correcting disease-causing mutations in the patient genome. As the most commonly used Cas proteins, SpCas9 and SaCas9 have been broadly applied in CVD-related modeling and therapeutic purposes, both in vitro [137–140] and in vivo [141–144]. Newly developed base editing and prime editing systems have also been used [145, 146]. Additionally, delivering CRISPR-Cas components to the cardiovascular system remains challenging, and AAV-based systems are currently the most widely used methods [147–150].

## Modeling CVD using CRISPR

Genetic studies have identified various pathogenic genetic variants associated with the occurrence of CVD [151, 152]. Revealing the consequences of specific mutations in CVD-related genes is important for CVD genetic diagnosis and precise medicine. CVD models have played a critical role in establishing causal links between genetic variants and CVD, dissecting the molecular mechanisms underlying CVD, validating therapeutic targets, and preclinical evaluation of therapeutic agents. At present, multiple gene editing tools have been applied to create in vitro and in vivo models of CVD [151].

### In vitro models of CVD

Human induced pluripotent stem cells (hiPSCs) are promising for modeling human cardiomyopathies in vitro because they can differentiate into cardiomyocytes [153]. Through genome editing of hiPSCs followed by their differentiation into cardiomyocytes (hiPSC-CMs), isogenic hiPSC-CMs have been broadly used to verify causative genes or mutations in cardiomyopathies. Gene disruption induced by CRISPR-Cas9 in hiPSC-CMs is straightforward and suitable for determining the role of a gene in CVD. For example, DNA methyltransferase 3A (*DNMT3A*) gene-deleted hiPSC-CMs generated using CRISPR-Cas9 gene editing showed altered contraction kinetics and impaired glucose/lipid metabolism, suggesting an important role of DNA methylation in cardiac diseases [137]. The homozygous *SCN10A* gene (encoding Na_V_1.8) knockout hiPSC-CMs help demonstrate that the voltage-gated sodium channel Nav1.8 contributes to late Na^+^ current (I_NaL_) formation and displays a harmful proarrhythmogenic function [138].

The precise introduction of point mutations into hiPSC-CMs facilitates the determination of the causal relationship between genetic mutations and heart diseases. Striated muscle-enriched protein kinase (SPEG) E1680K homozygous mutant hiPSC-CMs recapitulate the hallmarks of dilated cardiomyopathy (DCM), confirming that SPEG E1680K is a novel DCM-causing mutation [139]. Genome editing of hiPSCs has also been used to identify several causative mutations of arrhythmias. hiPSC-CMs expressing an R211H substitution in the Ras-related associated with diabetes (*RRAD*) gene mimic the single-cell electrophysiological characteristics of Brugada syndrome, a disorder predisposing the patient to ventricular arrhythmias, indicating that *RRAD* is possibly a novel susceptibility gene for Brugada syndrome [154]. CRISPR-Cas9-engineered hiPSC-CMs carrying three different mutations of ryanodine receptor 2 (RyR2), R420Q, Q4201R, or F2483I, exhibit various pathological features of catecholaminergic polymorphic ventricular tachycardia 1 (CPVT1)-associated arrhythmia, suggesting that different RyR2 mutations cause varied Ca^2+^ signaling consequences and drug sensitivities [155]. Genome-edited hiPSC-CMs can also be used as high-throughput platforms for scalable functional validation of the pathogenicity and pathophysiology of genetic variants identified in the human population. To determine the functional significance of cardiac troponin T (*TNNT2*) variants, the endogenous *TNNT2* gene was knocked out in hiPSCs using CRISPR-Cas9, and 51 different *TNNT2* variants were expressed using lentivirus in differentiated *TNNT2* knockout hiPSC-CMs. The results revealed that various *TNNT2* variants exhibit different pathogenic mechanisms, greatly expanding the knowledge of which and how *TNNT2* variants cause hypertrophic cardiomyopathy (HCM) and DCM [156].

Genome editing has been used to correct mutations to generate optimal isogenic controls for patient-derived iPSC-CMs, enabling the determination of genotype–phenotype relationships more precisely. Genome editing has been performed to correct the missense mutation T618I in the potassium channel gene *KCNH2* in short QT syndrome patience-specific hiPSC-CMs to elucidate the single-cell phenotype of short QT syndrome [157]. Using iPSC-CMs derived from doxorubicin-treated pediatric patients, cytosine base editing has helped identify the single nucleotide polymorphism rs11140490 in the SLC28A3 locus, which is a novel protector against doxorubicin-induced cardiotoxicity [145]. Isogenic hiPSC-CM controls generated by CRISPR-based gene correction have also been used as platforms to evaluate other therapeutic methods. Type 1 long QT syndrome is caused by loss-of-function variants in the KCNQ1-encoded Kv7.1 potassium channel α-subunit. iPSC-CMs generated from patients with KCNQ1-V254M and -A344A/spl mutations have recently been corrected using CRISPR-Cas9 to act as isogenic controls, which have been used to evaluate a dual-component suppression-and-replacement gene therapy method [158]. Base editing and prime editing could possibly be widely used in establishing hiPSC-CM-based CVD models in the near future.

### Animal models of CVD

CRISPR-based germline genome editing tools have revolutionized the generation of genetically modified animal models of CVD. Compared to conventional gene targeting technologies using embryonic stem cells, CRISPR-based gene editing technologies are easier to operate, faster, and applicable to most species. One strategy for generating animal models of CVD is to introduce targeted point mutations, insertions, or deletions using HDR-mediated germline genome editing. A mouse model of HCM with a *Myh6* R404Q mutation was generated using SpCas9/ssODN-mediated directed genomic DNA editing, and heterozygous mice developed a typical HCM phenotype [141]. A similar approach was also utilized to insert an additional adenine nucleotide into the lysosomal acid alpha-glucosidase (*Gaa*) gene at the c.1826 locus and generate a novel mouse model of infantile-onset Pompe disease (IOPD), which recapitulates HCM and the skeletal muscle weakness of human IOPD [142]. A CRISPR-Cas9-generated rat model, with a 9 bp deletion within the hotspot analogous to the novel mutation of the human PDE3A gene, recapitulates arterial hypertension with brachydactyly, demonstrating that mutant PDE3A causes arterial hypertension [143]. Recently, a 94 bp out of frame deletion was generated in exon 1 of *Kcnk3* using SpCas9/ssODN-mediated genome editing, creating a novel rat model of pulmonary arterial hypertension [159]. Another strategy is to delete exon(s) using two sgRNAs flanking specific exon(s). Exon deletion mutations in the dystrophin are among the most common causes of Duchenne muscular dystrophy (DMD). Several mouse models of DMD have been generated using CRISPR-Cas9 genome editing [144, 160], which are discussed further in the next section. CRISPR-Cas9 mediated mosaic inactivation of zebrafish *ccm2* led to a lethal multi-cavernous lesion that histologically mimics the typical human hemorrhagic cerebral cavernous malformation [161].

Compared with germline genome editing, somatic genome editing is a more flexible method for obtaining CVD models, which overcomes the challenges of germline modification, such as embryonic lethality and the cost and time required to establish, reproduce, and maintain these models. It is also suitable for rapid and relatively high-throughput studies on the functions of CVD-related genes. As early as 2016, a cardiomyocyte-specific SpCas9 transgenic mouse model was successfully generated to achieve somatic editing in the heart [162]. Following this study, intraperitoneal injection of AAV9 encoding sgRNA against three genes critical for the heart, *Myh6*, *Sav1*, and *Tbx20*, in postnatal cardiomyocyte-Cas9 transgenic mice caused a similar degree of DNA disruption and subsequent mRNA downregulation, but only *Myh6* disruption induced HCM and heart failure, suggesting that the effect of postnatal cardiac genome editing is target-dependent [147]. Mouse models can also be generated by activating endogenous gene expression through CRISPR-mediated genome editing in the postnatal heart [163]. CRISPR-mediated endogenous activation of myocyte enhancer factor 2D (*Mef2d*) leads to cardiac hypertrophy in mice, indicating that CRISPR-mediated genome editing can be used to generate CVD mouse models by controlling transcription in the postnatal heart [163]. Several recent studies have shown that CRISPR-Cas9 can be used to edit endothelial genes in vivo to obtain vascular disease models and enable reverse genetic studies of gene function in the mammalian vascular endothelium. Co-injection of an adenovirus harboring sgRNAs targeting the *Alk1* gene and AAV1-VEGF successfully induced mutations in *Alk1* in brain ECs and generated brain arteriovenous malformations in adult mice [164]. We recently generated a blood–brain barrier (BBB) breakdown mouse model by AAV-BR1-CRISPR mediated somatic genome editing. A single intravenous administration of brain microvascular EC targeting AAV-BR1 encoding sgRNA against the β-catenin (*Ctnnb1*) gene resulted in a mutation of 36.1% of the *Ctnnb1* alleles and dramatically decreased levels of CTNNB1 in brain ECs, leading to BBB breakdown in EC-restricted Tie2^Cas9^ mice [148]. The AAV-BR1-CRISPR system established in this study allowed for the rapid construction of BBB perturbation models in vivo and may be helpful for developing drug delivery systems in the central nervous system. Recently, the nanoparticle-mediated delivery of CRISPR plasmid DNA expressing Cas9 under the control of the *Cdh5* promoter resulted in efficient genome editing in the ECs of the peripheral vasculature in adult mice, which provides a powerful tool to construct animal models of peripheral vascular diseases [165].

## Genome editing in CVD treatment

Therapeutic genome editing can be used to treat monogenic CVD, and the technology could permanently correct mutations and eventually eradicate specific CVD. Programmed edits were introduced into the human germline genome [166–169]. However, human germline genome editing faces significant ethical concerns and is prohibited in most countries [170]. Somatic editing is a promising technology for editing CVD-causing mutations without the risk of passing genomic changes to the offspring. Table 1 summarizes the latest applications of genome editing in treating different types of CVD.

**Table 1:** Recent applications of genome editing in the treatment of CVD

Diseases	Models	Mutations	Strategies	Outcomes	References
DMD	Mice hiPSC-CMs	Deletion of *Dmd* exon44	Skipping and/or reframing of exon43 or 45 by single sgRNA targeting splice acceptor or donor site	Restore dystrophin expression and improve muscle function	[144]
	Mice hiPSC-CMs	Deletion of *Dmd* exon43, 45 or 52	Skipping and/or reframing of exon44 or 53 by single sgRNA targeting splice acceptor or donor site	Rescue the dystrophic phenotype, reduce necrotic cells and centralized nuclei	[171]
	Pigs hiPSC-CMs	Deletion of *Dmd* exon52	Deletion of exon51 by dual sgRNAs targeting flanking sequences	Improve muscle function and mobility and prevent malignant arrhythmias	[172]
	Mice	A nonsense mutation in *Dmd* exon23	Deletion of exon23 or 21–23 by dual sgRNAs targeting flanking sequences	Restore dystrophin expression and improve cardiac function without inducing serious adverse effects	[149, 150]
	Mice hiPSC-CMs	Deletion of *Dmd* exon51	Skipping of exon50 by destroying splice donor site using ABEmax in vivo; reframing of exon52 by PE in vitro	Restore dystrophin protein expression and normalize contractile abnormalities	[146]
	Mice	A 4 bp deletion in *Dmd* exon4	Skipping of exon4 by destroying splice donor site using CBE	Improve cardiac function and increase life span	[160]
	Mice	A nonsense mutation in *Dmd* exon53	Correction of the stop codon by ABE	A near complete rescue of dystrophin in hearts without obvious toxicity	[173]
HCM	Mouse embryos	A missense mutation in *Myh6* c.1211C > T (p. Arg404Trp)	Correction of the mutant nucleotide by ABEmax	Abolish HCM phenotypes in mice born from edited embryos	[141]
	hiPSC-CMs	Biallelic variants in *LZTR1* (paternal c.1943-256C > T create a new splice donor site and maternal c.27dupG)	Disruption of the SNP-induced splice donor site in the paternal allele by single sgRNA	Reverse the NS-associated hypertrophic phenotype including cell size, contractile and electrophysiological properties	[174]
	Human pronuclear stage zygotes	A dominant 4 bp deletion in *MYBPC3* exon16	HDR by microinjection of sgRNA, Cas9 protein and ssODN	Heterozygous gene mutations can be corrected in human gametes or early embryos	[175]
DCM	hiPSC-CMs	A 2 bp insertion in *TTN* exon327 c.70692_70693insAT	HDR by nucleofection of CRISPR/Cas9 RNP complex targeting the mutated allele and ssODN	Normalize titin protein level and contractile function	[140]
	hiPSC-CMs	A 1 bp deletion in *TTN* exon276	Reframing of the mutant allele by single sgRNA targeting the mutant site	Increase full-length TTN protein levels and sarcomere function	[176]
	hiPSC-CMs	Missense mutations in *RBM20* c.1901G > A (p. Arg634Gln) and c.1906C > A (p. Arg636Ser)	Correction of the mutant nucleotide by ABEmax and PE	Restore RBM20 nuclear localization and eliminate RNP granules in the cytoplasm	[177]
	Mice	A missense mutation in *Rbm20* c.1907C > A (p. Arg636Gln)	Correction of the mutant nucleotide by ABEmax	Restore RBM20 nuclear localization, eliminate RNP granules, restore cardiac size and function, and increase life span	[177]
Cardiac arrhythmia	Humanized mice	A 3 bp deletion in *PLN* c.40_42AGA (p.Arg14del)	Disruption of the mutant allele by single sgRNA targeting the mutant site	Improve cardiac function and reduce sustained ventricular tachycardia susceptibility	[178]
Atherosclerosis	Mice	–	Disrupting liver *Pcsk9* gene by adenovirus/AAV-delivered sgRNA targeting coding sequences	Disrupt *Pcsk9* genes and chronically decrease serum cholesterol levels	[30, 179]
	Mice macaques cynomolgus monkeys	–	Disruption of the splice donor site of liver *PCSK9* exon1 by AAV/LNP/eVLP-delivered ABE	Reduce plasma PCSK9 and LDL levels	[180, 181]

*DMD* Duchenne muscular dystrophy, *hiPSC-CMs* human induced pluripotent stem cell-derived cardiomyocytes, *sgRNA* single guide RNA, *ABE* adenine base editor, *PE* prime editor, *CBE* cytosine base editor, *HCM* hypertrophic cardiomyopathy, *SNP* single nucleotide polymorphism, *NS* Noonan syndrome, *HDR* homology-directed repair, *Cas9* CRISPR-associated nuclease 9, *ssODN* single-stranded oligodeoxynucleotide, *DCM* dilated cardiomyopathy, *CRISPR* clustered regularly interspaced short palindromic repeats, *RNP* ribonucleoprotein complexes, *AAV* adeno-associated virus, *LNP* lipid nanoparticle, *eVLP* engineered virus-like particle, *LDL* low-density lipoprotein

### DMD

DMD is an X-linked disorder characterized by proximal muscle weakness and cardiomyopathy caused by mutations in the largest human gene, dystrophin (*DMD*) [182]. A variety of mutations exist throughout the *DMD* gene, most of which are located in the regions crossing exons 43 to 53 and disrupt the ORF, resulting in non-functional truncated proteins.

A single-cut genome editing strategy was applied in both iPSC-CMs and mouse models of DMD bearing an exon 44 deletion mutation (Δ44), one of the most common causative mutations of DMD. The ORF can be restored by disrupting the exon splice site to skip the adjacent exon, inserting one nucleotide, or deleting two nucleotides in exon 44 [144]. Systemic delivery of gene editing components by a single dose of AAV9 restores ~ 90% dystrophin protein expression in the hearts of Δ44 mice within 4 weeks [144]. This approach also helps to correct DMD models bearing deletions of exons 43, 45, and 52 (Δ43, Δ45, and Δ52) both in vitro and in vivo [171]. A dual-AAV system was used to deliver SpCas9 and a single sgRNA targeting the splice donor site of exon 44 (for Δ43 or Δ45 mice) or splice acceptor site of exon 53 (for Δ52 mice) in vivo to restore dystrophin expression. Both exon skipping and reframing were induced in Δ45 and Δ52 mice, and the efficacy of dystrophin in these two models was higher than that in Δ43 mice, in which only exon skipping was generated [171]. Restoration of dystrophin has also been achieved in hiPSC-CMs from these DMD models [171]. However, this study did not specify whether exon skipping and/or reframing could subsequently rescue the cardiac phenotypes of DMD models [171]. Another strategy is to delete exon(s) using two sgRNAs flanking on either side, thus restoring the ORF of the *DMD* gene. The systemic application of AAV9 carrying an intein-split SpCas9 and a pair of sgRNAs targeting sequences flanking exon 51 in a pig model of DMD lacking exon 52 induced dystrophin expression in the heart and reduced arrhythmogenic vulnerability [172]. The long-term efficacy and safety of therapeutic editing for DMD have also been studied [149, 150]. AAV vectors carrying SaCas9 and a pair of sgRNAs targeting exon 23 or exons 21–23 were administrated for one year or 19 months, respectively, to mdx mice. Cardiac functions were improved without serious adverse effects, indicating that in vivo CRISPR genome editing may be a safe therapeutic strategy for DMD [149, 150].

Base editing and prime editing show great promise for treating DMD. Both ABE and PE can restore dystrophin protein expression by inducing exon skipping or exon reframing to correct the *Dmd* exon 51 deletion mutation in iPSC-CMs, and intramuscular delivery of AAV9 encoding ABE components amends the mutation in ∆E51 DMD mice [146]. CBE has been shown to rescue dystrophic cardiomyopathy in *Dmd*^E4*^ mice, which harbor a 4 bp deletion in exon 4 of the *Dmd* gene and recapitulate many characteristics of human DMD [160]. A single-dose administration of AAV9-eTAM encoding a fused nuclease-defective SaCas9 (KKH) with activation-induced cytidine deaminase (AID) and UGI, together with AAV9-sgRNA, efficiently induced splice site mutation and exon 4 skipping of the *Dmd* gene and restored up to 90% of dystrophin proteins in the heart of *Dmd*^E4*^ mice, resulting in improved cardiac function and an increased life span [160]. Alternatively, a dual AAV-mediated protein trans-splicing approach was used to deliver a modified ABE-NG to an *mdx*^4cv^ mouse model carrying a premature stop codon (CAA-to-TAA) in exon 53 of the *Dmd* gene. After 10 months of treatment, a near-complete rescue of dystrophin was found in the hearts of *mdx*^4cv^ mice without obvious toxicity [173].

### HCM and DCM

Inherited cardiomyopathies, including HCM and DCM, are candidate genetic disorders that are suitable for genome editing-related treatment. ABEmax-NG has been shown to correct a pathogenic R404Q/+ mutation in embryos of the HCM mouse model [141]. Administration of ABEmax-NG mRNA to *Myh6*^R404Q/+^ embryos corrects the mutant allele at a rate of 62.5% to 70.8%, abolishing the HCM phenotype in postnatal mice and their progeny. Moreover, in utero delivery of intein-split ABEmax-NG induced a high correction rate without introducing indels or off-target editing in *Myh6*^R404Q/+^ fetuses [141]. Intronic CRISPR repair has been demonstrated as efficient in a preclinical iPSC-CM model of Noonan syndrome-associated HCM [174]. CRISPR-Cas9-mediated destruction of the mutation-induced additional intronic donor splice site can reverse the hypertrophic phenotypes in Noonan syndrome patient-derived iPSC-CMs carrying biallelic mutations in intron 16 of the leucine zipper-like transcription regulator 1 (*LZTR1*) gene, indicating new possibilities for personalized therapeutic genome editing in HCM patients [174]. Notably, CRISPR-based genome editing has been shown to have potential to correct a well-documented heterozygous dominant 4 bp deletion in exon 16 of *MYBPC3*, which causes familial HCM, in human embryos [175]. Co-injection of Cas9 proteins, mutation-specific sgRNAs, and mutant sperm into healthy metaphase II oocytes corrected the deletion by wild-type maternal allele-mediated HDR, resulting in a high yield of homozygous embryos carrying the wild-type *MYBPC3* gene without mosaicism or off-target mutations [175].

Genome editing has also been used to correct DCM-causing mutations. In addition to previous studies using hiPSC-CMs showing that truncated titin (TTNtv) mutations are the most common causes of DCM [183–185], recently, the pathological mechanisms of TTNtv-associated DCM have been highlighted and a new genome editing strategy has been developed to treat TTNtv-associated DCM. iPSC-CMs with patient-derived or CRISPR-Cas9-generated *TTN* mutations were corrected using SpCas9/ssODN. Engineered heart muscle generated from corrected hiPSC-CMs shows normalized titin protein levels and contractile function [140]. Genome editing using SpCas9 and A-band *TTNtv*-specific sgRNA was also shown to restore the reading frame of TTN protein in hiPSC-CMs, leading to increased full-length TTN protein levels and normalized sarcomere function [176]. More recently, the application of precise genome editing technology for treating DCM caused by mutations in *RBM20* has been reported [177]. The RBM20^R634Q^ and RBM20^R636S^ mutant iPSCs were corrected by ABE and PE, with the efficiency of 92% and 40%, respectively. In addition, AAV9-mediated systemic delivery of ABE components corrected 66% of the *RBM20* transcripts expressed in cardiomyocytes of postnatal RBM^R636Q/R636Q^ mice. The corrected mice showed restored cardiac size and function, and prolonged life span [177].

### Cardiac arrhythmia

Cardiac arrhythmia caused by autosomal-dominant mutations can be treated with CRISPR-mediated specific disruption of the mutant allele, which has been validated in several mouse models [186, 187]. Recently, humanized mice expressing a human mutant PLN (hPLN-R14del) demonstrated bi-ventricular dilation and a higher propensity for sustained ventricular tachycardia. Disruption of the hPLN-R14del allele by AAV9-CRISPR-Cas9 improved cardiac function and reduced sustained ventricular tachycardia susceptibility in young adult humanized PLN-R14del mice, providing a potential therapeutic strategy for the arrhythmogenic phenotype in human patients with the PLN-R14del mutation [178].

### Atherosclerosis

Atherosclerosis is a chronic disease that refers to the formation of fibrofatty lesions in the arterial wall, and causes ischemic stroke, ischemic cardiomyopathy, myocardial infarction, and peripheral arterial disease. Blood concentration of low-density lipoprotein cholesterol (LDL-C) is one of the best-established causal risk factors for atherosclerosis.

The lipid metabolism-related gene, *Pcsk9*, is specifically expressed in the liver and functions primarily as an antagonist to the LDL receptor. Disruption of PCSK9 activity can reduce circulating LDL-C levels, thereby lowering the risk of atherosclerosis [188]. Several clinical trials have investigated monoclonal antibodies targeting PCSK9. However, even if these antibody-based drugs are effective, their effect on LDL-C is short-lived. Genome editing using CRISPR systems provides an alternative method for reducing PCSK9 levels. A single administration of adenovirus co-expressing SpCas9 and sgRNA targeting exon 1 of the mouse *Pcsk9* gene can efficiently introduce loss-of-function mutations into endogenous *Pcsk9* genes in vivo and chronically decrease plasma cholesterol levels in the blood [179]. The AAV-SaCas9 system has also been proven to be effective in editing the *Pcsk9* gene in vivo, leading to significantly decreased serum PCSK9 and cholesterol levels [30]. Single injections of engineered DNA-free VLPs targeting the *Pcsk9* gene into adult mice demonstrated 63% base editing in the liver, resulting in 78% reduction in serum PCSK9 levels [126]. In addition to these studies carried out in rodents, somatic *Pcsk9* gene editing has also been validated in nonhuman primates [180, 181]. CRISPR base editors delivered using LNPs proved highly effective in editing the *Pcsk9* gene in the liver of macaques and cynomolgus monkeys. A single-dose treatment of LNPs carrying CRISPR base editors leads to stable *Pcsk9* knockdown in the liver and a 60% reduction in blood LDL-C for at least 8 months [180]. To ensure the safety of gene editing in comparatively more proliferative organs, such as the liver, the proportion of edited cells that remain stable over time must be investigated. All these genome editing approaches offer the potential for once-and-done therapies for the lifelong treatment of atherosclerosis-associated CVD.

## Perspectives

CRISPR-based genome editing technology has been rapidly applied in almost all fields, from basic biology to translational medicine. The development of novel systems and tools for more accurate, efficient, and faster genome editing and tighter control of the duration, efficiency, and specificity of genome editors will further benefit their translational applications. Newly uncovered thousands of phage-encoded CRISPR systems provide a valuable resource for searching novel miniature single-effector CRISPR-Cas systems [39]. In addition, newly developed Cas13a-based RNA editing tools can achieve RNA knockdown and precise base editing of mammalian transcripts without causing DNA damage, providing a promising potential therapeutic strategy in translational cardiovascular medicine [11, 189]. Notably, type III-E CRISPR-Cas7-11 effector has recently been shown to cleavage protein under target RNA guidance [190, 191], bringing new potential CRISPR tools for CVD diagnosis and treatment.

Genome editing technologies have been successfully translated into human clinical trials for enhanced chimeric antigen receptor (CAR) T-cell therapy, cell-based regenerative medicine, and treatment of monogenic diseases, such as transfusion-dependent beta thalassemia (TDT) and sickle cell disease (SCD) [192, 193]. Researchers have used in vivo genome editing to target the transthyretin (TTR) gene to treat transthyretin amyloidosis and have achieved very encouraging results in phase 1 clinical trials, taking the most critical step towards applying CRISPR-based genome editing technology to treat human genetic diseases [123]. Taking the most optimistic view, CVD with known causal genes can theoretically be treated with CRISPR technology. However, there are still several important challenges. Recently, CRISPR-based genome editing in human embryos was shown to cause unpredictable genomic alterations, including DNA rearrangements, large deletions, and even loss of allele-specific chromosomes [168, 194, 195]. Therefore, potential technical safety concerns, including mosaicism, off-target effects, and long-term risks caused by genome editing, need to be addressed before the therapeutic applications of CRISPR technology in treating CVD. Perhaps striking a balance between the efficiency and safety of genome editing is crucial. At present, efficient delivery of CRISPR-Cas systems to human cardiovascular system remains a challenge. In addition, the efficacy and safety of each therapeutic gene editing strategy for each CVD need to be confirmed by clinical trials. Although this paper uses the CVD as example to illustrate the progress of CRISPR-based genome editing in modeling and treating diseases, the same strategies could also be used for numerous diseases in other tissues.

Like any cutting-edge technology, gene editing technology could be a double-edged sword. Genome editing has been listed as a potential weapon of mass destruction in the 2016 annual worldwide threat assessment report of the U.S. intelligence community, indicating a high risk of extreme misuse. Recent rapid advances have made genome editing technologies more accessible and difficult to control, which may further lower the threshold for genome editing misuse and increase biosecurity threats. The possible misuse of genome editing technology and biosecurity risks may include, but are not limited to creating (1) pathogens with increased virulence, (2) new pathogens and biotoxins, and (3) gene-driven animals that may have irreversible effects on specific populations and the environment. Regulations and guidelines should be developed after extensive consultation to ensure that the development of gene editing technologies will not harm living organisms, including humans, or the environment.

## Conclusions

The emerging novel Cas nucleases and their extended applications have greatly expanded the CRISPR-based genome editing toolbox and promoted the development of life science and medicine. CRISPR-based genome editing technology has also revolutionized cardiovascular research, accelerating the generation of genetically modified models of CVD and its application in the treatment of different types of CVD. However, this technology may also bring huge potential biological threats, which should be strictly controlled to prevent its abuse.

## Data Availability

Not applicable.

## Abbreviations

AAV: Adeno-associated virus
ABE: Adenine base editor
BBB: Blood–brain barrier
CRISPR: Clustered regularly interspaced short palindromic repeats
Cas: CRISPR-associated protein
CVD: Cardiovascular disease
Cascade: CRISPR-associated complex for antiviral defense
crRNA: CRISPR RNA
CBE: Cytosine base editor
CGBE: C-to-G base editor
CAST: CRISPR-associated transposon
CAR: Chimeric antigen receptor
DCM: Dilated cardiomyopathy
DMD: Duchenne muscular dystrophy
DSB: Double-strand break
EC: Endothelial cell
FiCAT: Find and cut-and-transfer
hiPSC: Human induced pluripotent stem cell
HCM: Hypertrophic cardiomyopathy
HDR: Homology-directed repair
LNP: Lipid nanoparticle
LDL-C: Low-density lipoprotein cholesterol
M-MLV: Moloney murine leukemia virus
NHEJ: Non-homologous end-joining
ORF: Open reading frame
OMEGA: Obligate mobile element-guided activity
PAM: Protospacer adjacent motif
PE: Prime editor
pegRNA: Prime editing gRNA
RNP: Ribonucleoprotein complexes
sgRNA: Single guide RNA
ssODN: Single-stranded oligodeoxynucleotide
SCD: Sickle cell disease
tracrRNA: Trans-activating CRISPR RNA
TAM: Transposon-associated motif
TDT: Transfusion-dependent beta thalassemia
UGI: Uracil-DNA glycosylase inhibitor
UNG: Uracil-DNA glycosylase
VLP: Virus-like particle
